# Perinephric Hematoma with Active Arterial Hemorrhage following Extracorporeal Shockwave Lithotripsy

**DOI:** 10.1155/2019/1547437

**Published:** 2019-01-06

**Authors:** Edward Assaf, Rawad Abou Zahr, Elie Ghabi, Imad Ghantous

**Affiliations:** ^1^Urology Department, St George Hospital University Medical Center, University of Balamand, Beirut, Lebanon; ^2^Faculty of Medicine and Medical Sciences, St George Hospital University Medical Center, University of Balamand, Beirut, Lebanon

## Abstract

Subcapsular hematoma is an exceedingly rare complication of extracorporeal shockwave lithotripsy (ESWL) for renal stones with cases demonstrating evidence of active arterial bleeding even more so. A 49-year-old male presented with acute onset right flank pain two hours following ESWL. CT scan with contrast revealed active contrast extravasation consistent with arterial bleeding. The patient was managed with arterial embolization and recovered uneventfully following a 4-day hospitalization.

## 1. Introduction

Since its first application by Chaussy et al., extracorporeal shockwave lithotripsy (ESWL) has been a safe, noninvasive, and successful technique for the management of renal calculi [1]. Complications, however, are possible, range from benign and self-limited to catastrophic, and have been recognized for several years [2, 3]. Renal hematoma is one such injury [3–11]. Ultrasound screening reveals an incidence of renal hematoma formation post-ESWL ranges from 0.1% to 0.6% [4], whereas magnetic resonance (MR) screening reveals an incidence of 20–25% [3]. The incidence of clinically significant hematoma is reported to be <1%; however, newer generation imaging modalities determine its ranges from 3% to 12% [9]. Documented cases of renal hematoma following ESWL were benign and self-limited, successfully managed with conservative therapy [4–8] or percutaneous drainage [10, 11]. Only few cases of active arterial hemorrhage (reported by Silberstein [9] among others) exist in the literature, making the occurrence exceedingly rare in an already rare phenomenon. The following case report is of a 49-year-old patient who developed perinephric hematoma with evidence of active arterial extravasation on computed tomography (CT) scan and arterial angiography that was successfully treated with arterial embolization and follow-up supportive care. By highlighting this occurrence, we hope to increase clinical suspicion of active bleeding when patients present with acute onset flank pain following EWSL.

## 2. Case Presentation

**Figure 1:**
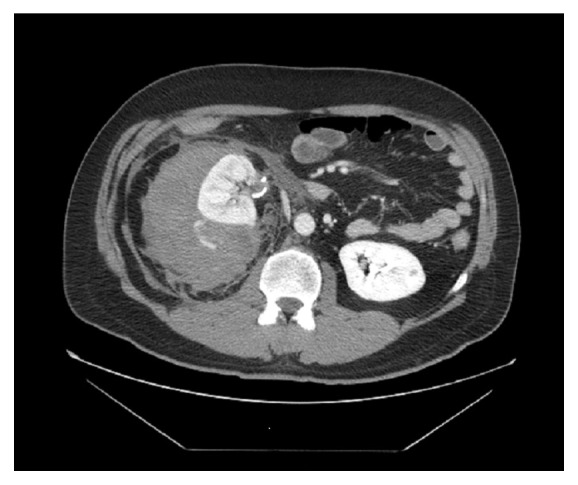
Active contrast extravasation from the right kidney on computed tomography.

**Figure 2:**
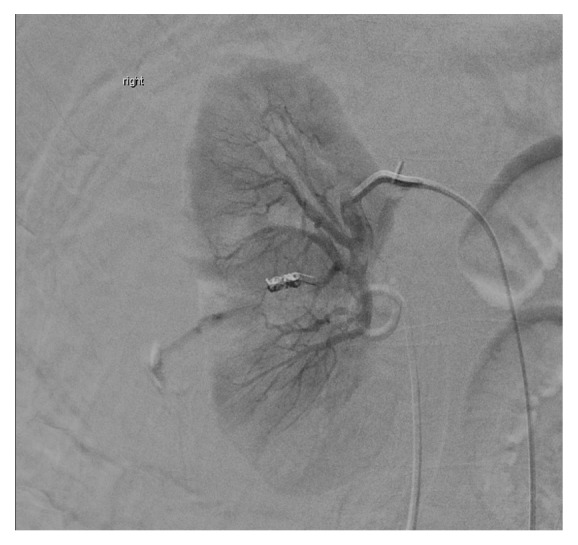
Active contrast extravasation on arteriography.

**Figure 3:**
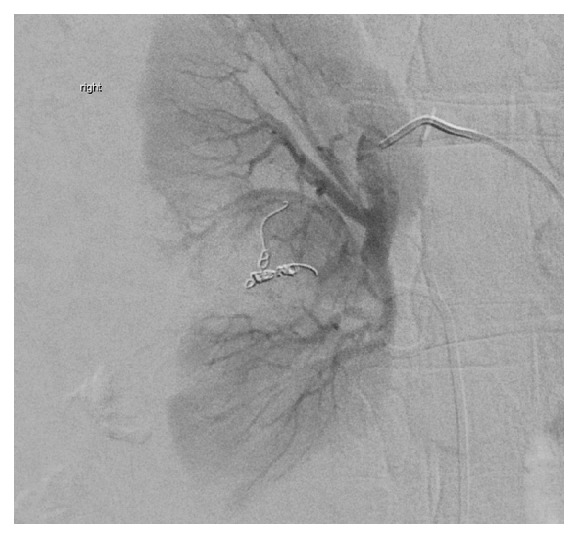
Successful arterial embolization demonstrating no signs of active contrast extravasation.

A 49-year-old male presented to our hospital for severe pain in his right flank of 2 hours duration following Extracorporeal Shockwave Lithotripsy (ESWL) performed on the same day for a stone in his right kidney. His past medical history includes nephrolithiasis, for which he underwent ESWL 3 years prior to presentation. At presentation, the patient was afebrile and hemodynamically stable with normal vital signs. Examination revealed exquisite tenderness over the right costovertebral angle. Laboratory studies revealed a hemoglobin value of 14.9g/dL, a hematocrit of 43.9%, creatinine of 1.03 mg/dL, and BUN of 30 mg/dL. An abdominal CT with contrast revealed 17x13x11 cm right perinephric hematoma with evidence of active contrast extravasation in the arterial phase (Figure 1). A decision was made to send the patient for emergent arteriography. Catheterization was performed with a 5F Cobra catheter and contrast injection revealed active extravasation arising from the small branches of the middle subsegmental posterior renal artery (Figure 2). The subsegmental branches were cannulated using a 2.7F Cobra catheter, and contrast injection revealed two bleeding branches from the posterior capsule. Embolization with 0.018 microcoils was performed and subsequent contrast injection did not show evidence of extravasation (Figure 3). The patient was admitted for monitoring and supportive care. Serial complete blood count revealed a steady decline of hemoglobin concentration to reach 9.3g/dl on the 3^rd^ day of hospitalization. At the time, the patient had nausea, fatigue, pallor, and tachycardia. He was successfully managed with transfusion of 1 unit of packed red blood cells and IV hydration. A follow-up CT scan revealed a slight reduction in the size of the hematoma to measure 16x12x10cm. Throughout his hospitalization, the patient's renal function was intact as determined by a stable creatinine value of 0.8 mg/dL (estimated GFR = 105ml/min/1.73m^2^ as calculated by CKD-EPI). Following hemodynamic stabilization and symptom resolution, the patient was discharged after a 4-day hospitalization and was followed up on an outpatient basis.

## 3. Discussion

Though major complications of ESWL are rare, several complications are frequently observed, most commonly microhematuria due to microtrauma to the kidney. Other complications include acute pyelonephritis, acute kidney failure, and ureteric obstruction due to stone fragments [3]. Nonrenal complications also occur with reports of myocardial injury, abdominal aneurysmal rupture, venous thrombosis, and subcapsular hepatic hematoma, though these occurrences are rare [3, 9, 12]. Renal subcapsular and perinephric hematoma is a recognized complication with an incidence ranging from 0.1 to 0.6% as detected by ultrasound [3] and between 20 and 25% as detected by MR or CT [4]. A retrospective study between 1987 and 1996 revealed the incidence of renal hematoma to be 0.28% relative to the number of patients (10,953) and 0.14% relative to the number of sessions (21,699) with the most common presenting symptom (74%) being lower back pain [4]. Several risk factors were found to be predispose to renal hematoma formation, particularly preexisting hypertension [9]. Knapp et al. also report that the incidence of hematoma increased from 0.66% to 2.5% in hypertensive patients and to 3.8% in patients with poorly controlled hypertension [13]. Furthermore, a multivariate analysis revealed no association between mean arterial blood pressure and the incidence of renal hematoma [9]. Other risk factors include diabetes mellitus, atherosclerosis, obesity, bleeding diathesis, male gender, and age with a 1.67 times increased risk for every 10-year increase in age [3, 4, 9]. Moreover, the number of shocks and frequency of shock delivery were found to be a significant predisposing factor to hematoma formation [9]. Patient presentation varies, though the most common complaints at presentation were lower back pain and flank pain. Gross hematuria is a common complaint, usually significant of renal injury but not to the extent of renal damage. Patients presenting with gross hematuria were found to have benign renal injury and impairment that resolved within 12 hours [9]. Patients who develop hematoma are generally managed conservatively; however a high index of suspicion for active bleeding, renal or otherwise, should be present.

In our case, the patient presented with flank pain, a relatively common symptom after ESWL, and he was hemodynamically stable. However, the severity of the pain along with nonresponsiveness to analgesia raised the suspicion of a more severe pathology, thus warranting further investigation by CT scan.

It is not common practice to order CT scans for every patient presenting for flank pain after ESWL, since most of these patients will respond to analgesia and the pain is usually a classic renal colic type pain.

## 4. Conclusion

Renal hematoma following ESWL for renal calculi is a rare but generally benign condition only requiring conservative management. Few serious complications occur requiring more invasive therapy. A hematoma due to an active arterial bleed is exceedingly rare but should be suspected in patients presenting with lower back or flank pain acutely after ESWL, especially in patients who are unresponsive to intravenous analgesics. Active bleeding, though rare, should be considered despite hemodynamic stability, thereby warranting further investigation.
